# Single-Stage Arthroscopic Cartilage Repair With Chondrectomy and Implantation of a Templated Membrane Collagen Scaffold With Bone Marrow Aspirate Concentrate Augmentation (AMIC Plus)

**DOI:** 10.1016/j.eats.2023.07.030

**Published:** 2023-10-30

**Authors:** Zachariah Gene Wing Ow, Katrina Jia En Ting, Keng Lin Wong

**Affiliations:** a Department of Orthopaedic Surgery, Sengkang General Hospital, Singapore; b Musculoskeletal Sciences Academic Clinical Programme, Duke–NUS Graduate Medical School, Singapore

## Abstract

Single-staged cartilage repair techniques have shown great clinical efficacy in the treatment of articular cartilage defects of the knee, particularly when using bilayered acellular scaffolds augmented with bone marrow aspirate concentrate. We describe an all-arthroscopic approach to the single-staged cartilage repair procedure using a porcine-derived collagen I/III bilayered scaffold that is templated arthroscopically and augmented with bone marrow aspirate concentrate, in the treatment of critically sized articular defects of the knee.

Single-staged cartilage repair procedures have shown comparable outcomes with staged procedures such as autologous chondrocyte implantation, with a crucial benefit of being performed in a single surgery, often at a lower overall cost and with greater international regulatory acceptance. At the forefront of single-staged cartilage repair is the AMIC procedure, wherein an acellular collagen I/III bilayered scaffold, Chondro-Gide (Geistlich Pharma AG, Wolhusen, Switzerland) is used with or without bone marrow aspirate concentrate (BMAC), with the latter being termed AMIC Plus. Chondro-Gide has been shown to provide the greatest degree of pain relief in short-term follow-up, with BMAC augmentation providing further improvement of outcomes.^1^ Cartilage repair with Chondro-Gide traditionally has been performed through a mini-open approach through a parapatellar incision to allow for adequate lesion exposure, and templating and fixation of the acellular patch. However, in this article, we present our technique of an all-arthroscopic cartilage repair with the AMIC Plus procedure.

## Surgical Technique (With Video Illustration)

### Patient Evaluation, Imaging, and Indications

Careful and comprehensive preoperative evaluation of patients presenting with symptomatic articular cartilage lesions is of paramount importance.^2^ Apart from localizing the likely areas of cartilage defects through clinical examination, identifying possible concomitant pathologies such as ligamentous damage, malalignment, and rotational deformities is crucial, as such comorbidities could contraindicate upfront cartilage repair. Standard weight-bearing orthogonal views of the knee joints, as well as sunrise or skyline Merchant views, in addition to long-leg films are valuable diagnostic tools and should be performed in the same initial clinic consultation; however, the gold-standard imaging modality for soft-tissue injuries of the knee still remains magnetic resonance imaging. Indications for cartilage repair include symptomatic lesions that affect activities of daily living or sporting activities, which have failed conservative management such as physiotherapy and intra-articular injections. Cartilage defects that are eligible for repair should be contained defects with healthy cartilage surrounding the defect.

### Patient Positioning and Anesthesia

Under general anesthesia, the patient is positioned supine on the operating table with the ipsilateral foot attachment removed, allowing for a full range of motion, and improved access to the knee joint. A high thigh tourniquet is then placed and inflated.

### Diagnostic Arthroscopy

**Fig 1:**
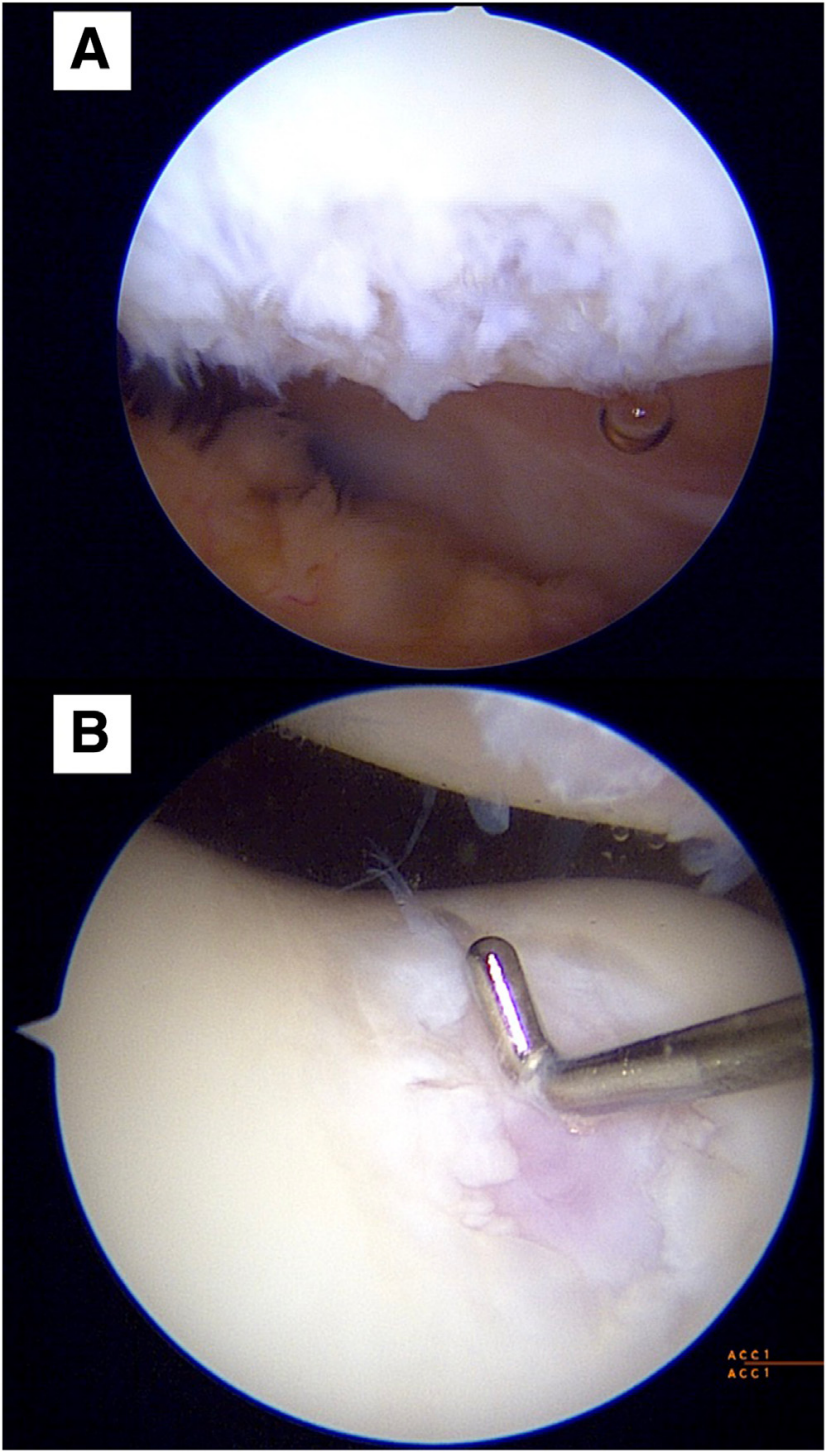
Direct arthroscopic visualization of the articular defects is obtained. Here, we present an Outerbridge IV articular cartilage defect of a patient’s right patella (A), as well as an Outerbridge IV articular cartilage defect of another patient’s right trochlea, with probe seen for measurement of the defect (B). Both defects were viewed via the anterolateral portal.

With reference to the surface marking of the patella tendon, standard anteromedial and anterolateral portals are created, and the joint space is instilled with normal saline. Diagnostic arthroscopy is performed with a 30° arthroscope to directly visualize, probe, and stage the cartilage lesion. A note is made of any concomitant soft-tissue injuries (Fig 1).

### Preparation of AMIC Plus: Chondro-Gide and BMAC

**Fig 2:**
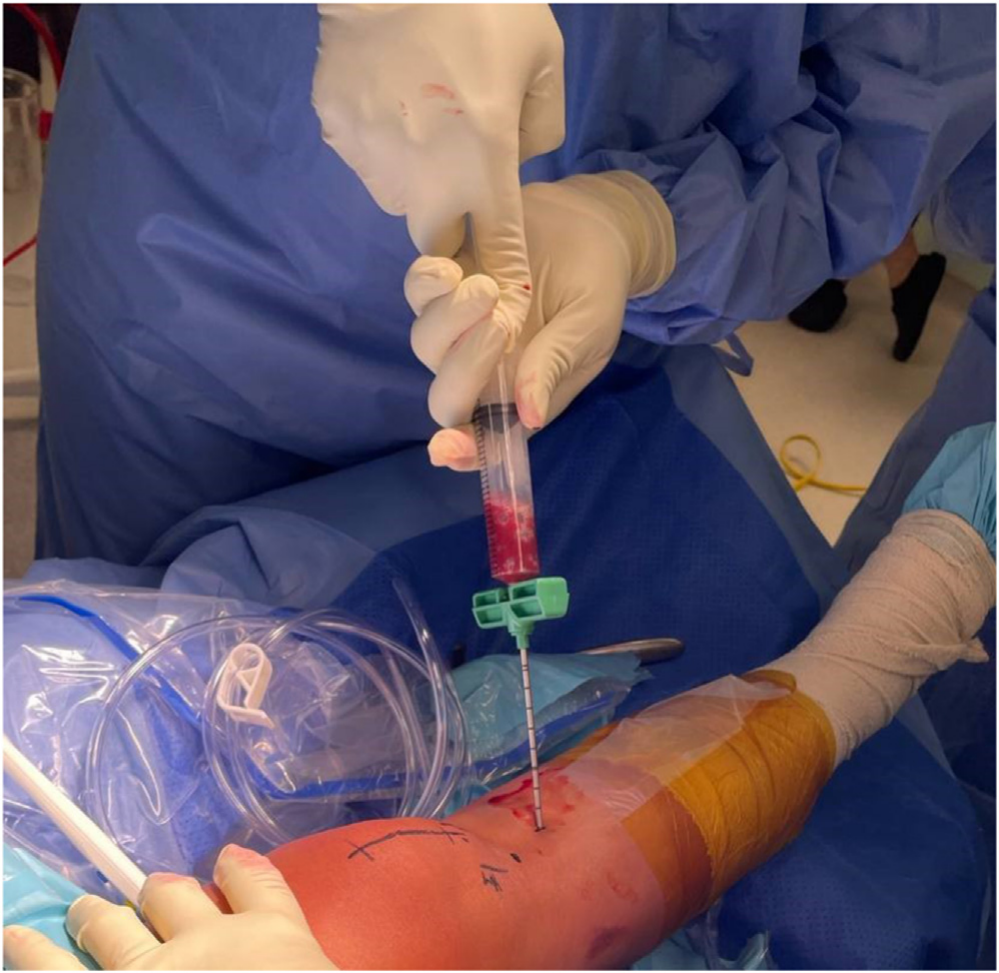
Harvesting of bone marrow aspirate is performed using a trephine bone marrow–aspiration device (BioCUE BMA) at the ipsilateral right proximal tibia.

A bone marrow aspiration trocar is inserted to the ipsilateral proximal tibia, and 30 mL of bone marrow is aspirated using the BioCUE BMA Concentration System (Biomet Biologics, Warsaw, IN) as per manufacturer guidelines. The aspirate is then centrifuged at 3200 rpm for 15 minutes with the GPS III system (Zimmer Biomet, Warsaw, IN) to produce 3 to 5 mL of BMAC (Fig 2).

**Fig 3:**
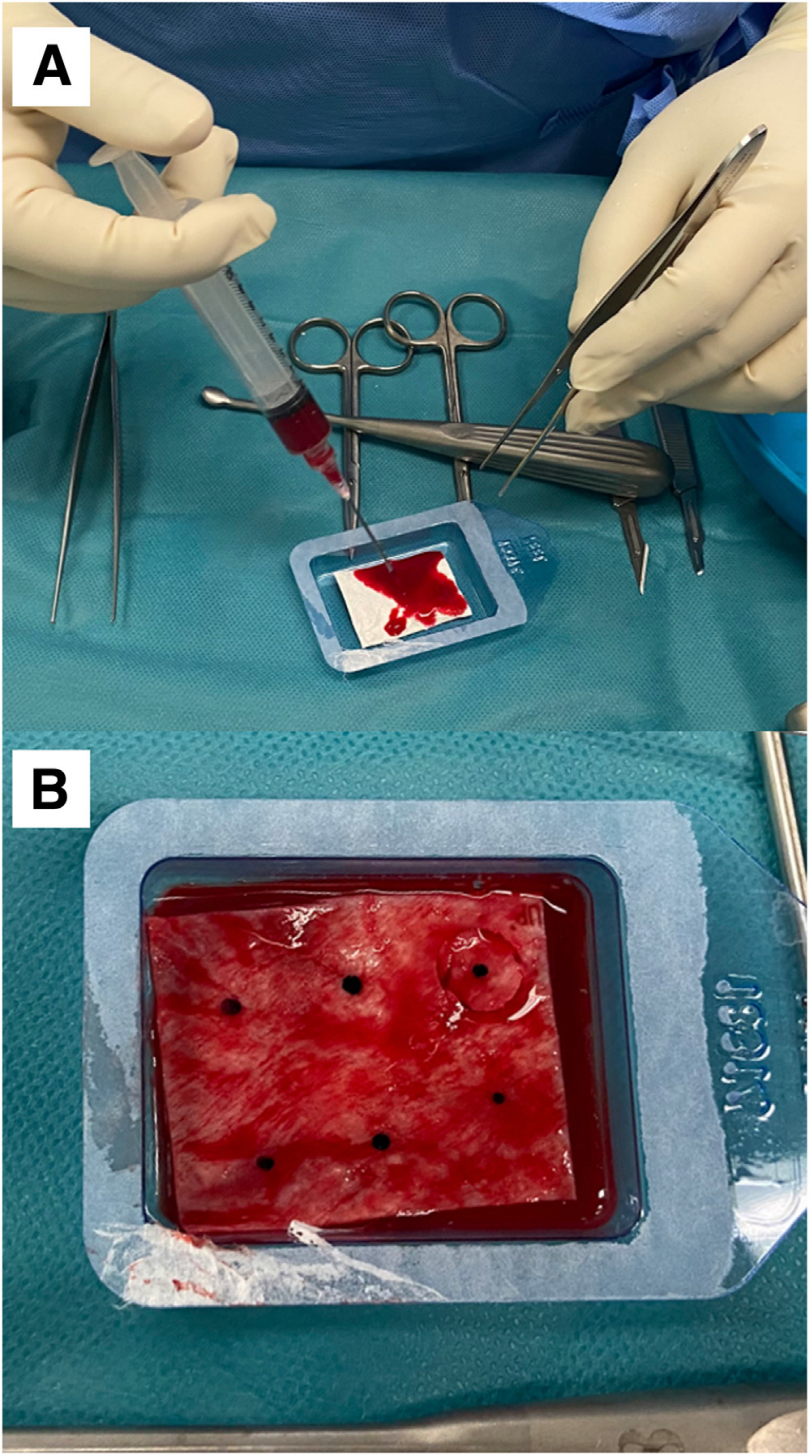
(A) BMAC is instilled into the Chondro-Gide scaffold, ensuring adequate soaking of the graft. (B) Using a sterile skin marker, the articular surface of the Chondro-Gide scaffold is marked for easier reference during arthroscopic implantation.

The sterile package cover of collagen I/III bilayer membrane scaffold, Chondro-Gide, (Geistlich Pharma AG) is opened, the articular surface marked with a sterile skin marker, and the BMAC solution instilled to the Chondro-Gide sheet within the sterile package container and left to soak for 5 minutes (Fig 3).

### Cartilage Lesion Preparation

**Fig 4:**
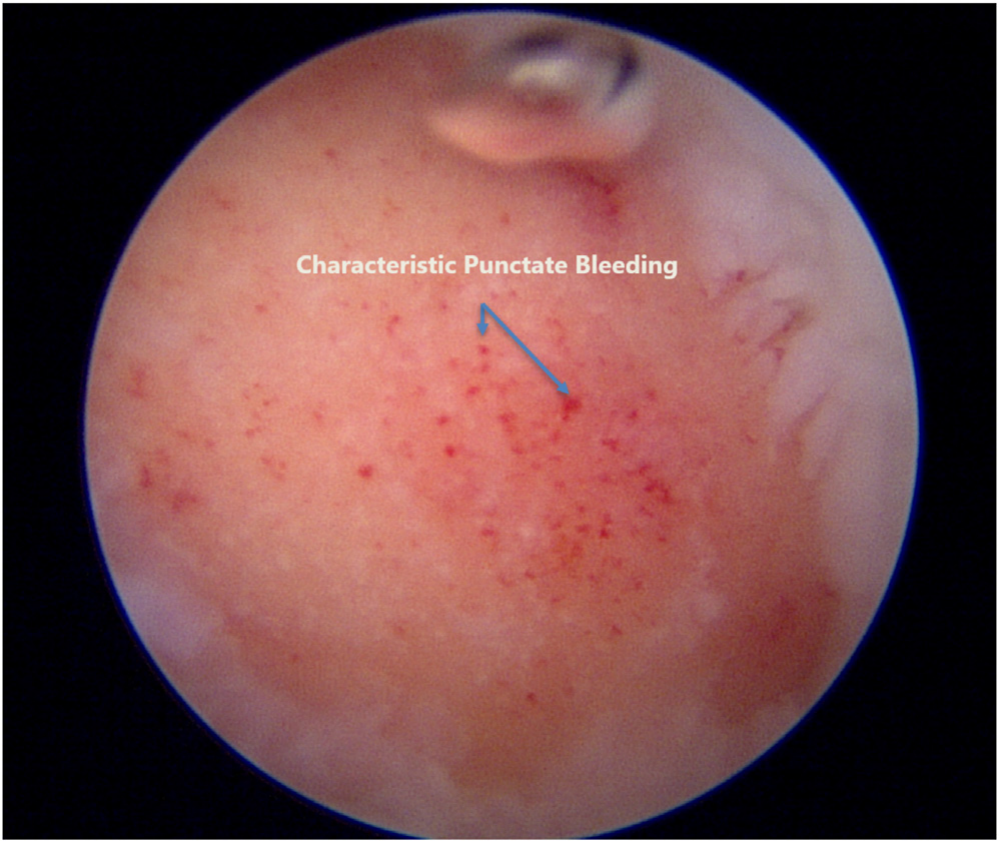
Characteristic punctate bleeding on slowly reducing saline instillation pressure, signifying an adequately debrided lesion bed.

**Fig 5:**
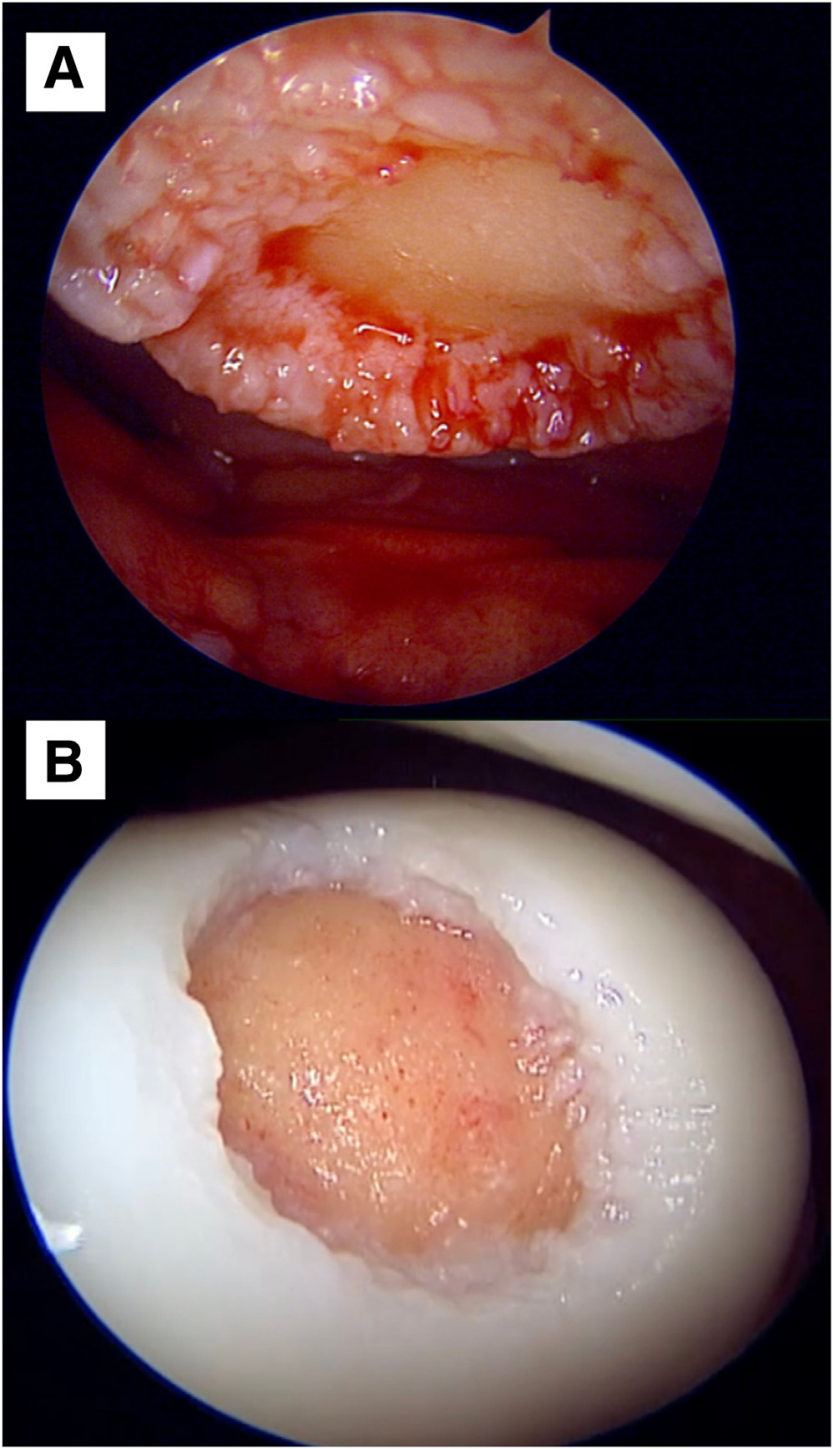
Cartilage defects, previously shown in Fig 1, now debrided. Dry arthroscopy via carbon dioxide insufflation is now used to prepare for Chondro-Gide implantation. (A) Right knee patella cartilage defect post debridement, measuring 1.5 cm by 1.5 cm. (B) Right knee trochlea cartilage defect post debridement, measuring 2 cm × 2 cm.

The Chondrectom Extended set – big joints (Biovico, Gdynia, Poland) is used to debride and prepare the cartilage defect site for graft implantation. The front and sideward chondrectomes are used to make sharp, 90° cuts of the vertical lesion margins, whereas the parallel chondrectome is used to grossly debride the defect bed and remove any intralesional osteophytes. Finer preparation of the defect bed is achieved by using a curette to gently debride the bed to the level of the articular tidemark, using audiological and tactile cues to signal an appropriate debridement.^3^ The instillation pressure of the saline arthroscopy is reduced, and the lesion base is observed for characteristic punctate bleeding, signifying an adequate depth of debridement (Fig 4). No microfracture is performed for the defect bed. At this juncture, saline instillation is halted, and the procedure is converted to a dry arthroscopy after suctioning of the remaining saline and instillation of carbon dioxide gas to the knee (Fig 5).

### Graft Templating

**Fig 6:**
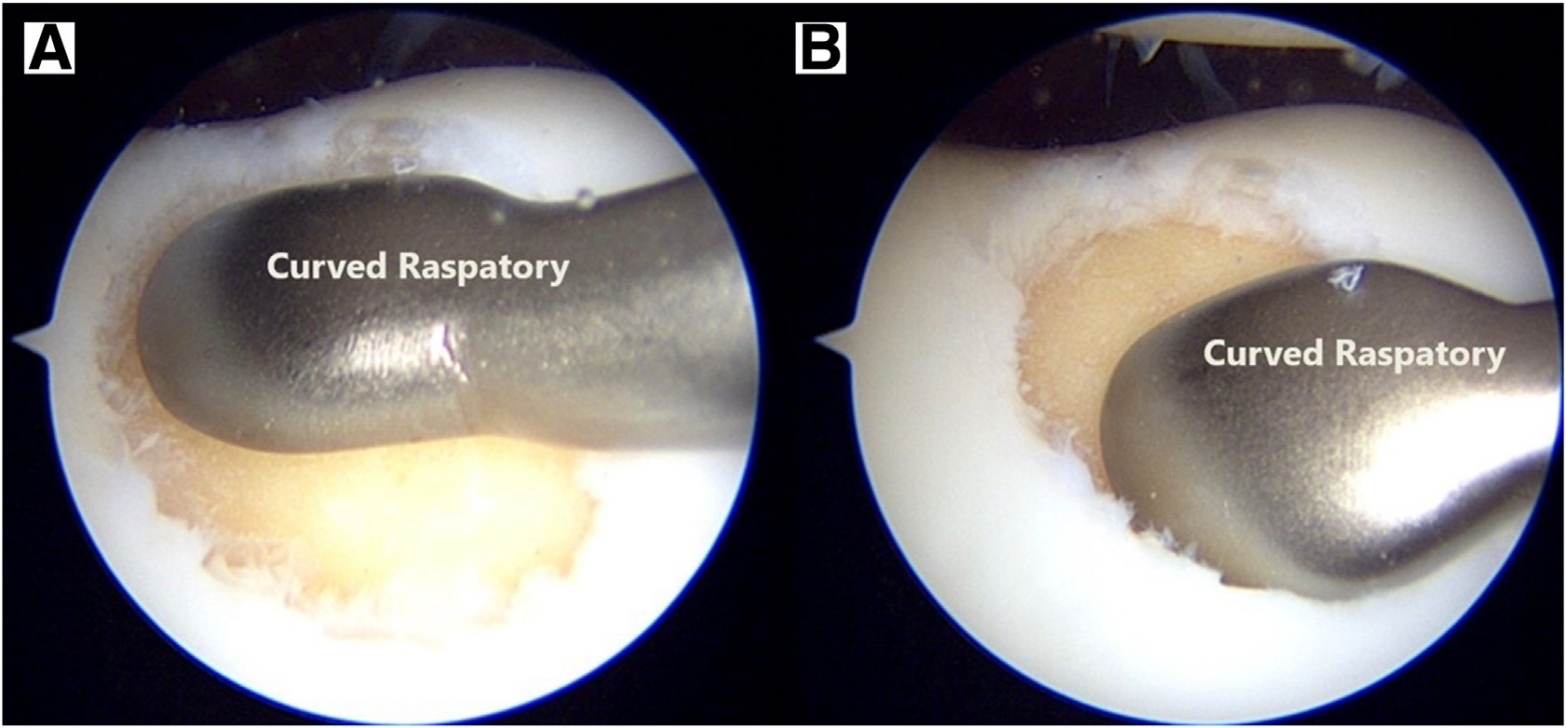
Estimating the graft size required to fill the lesion bed by using a curved raspatory tool. (A) Superior margin. (B) Inferior margin.

**Fig 7:**
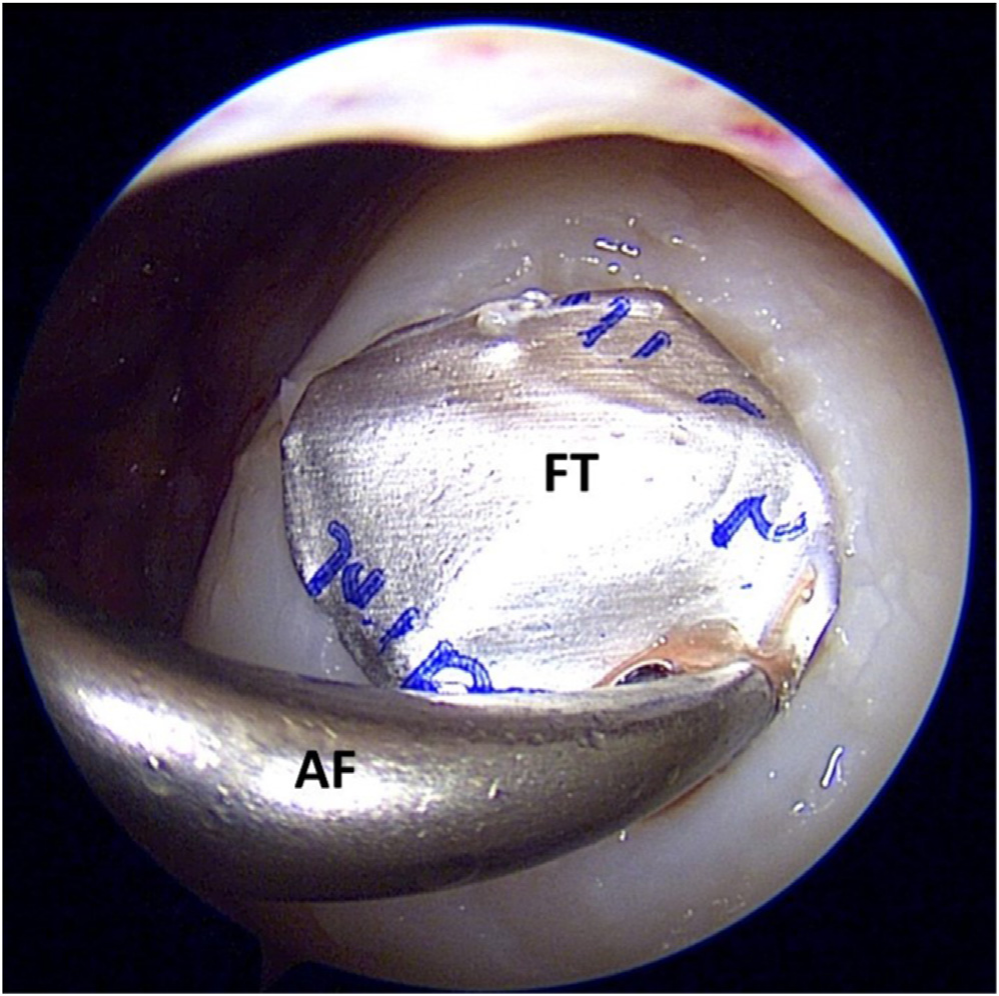
Using a pair of artery forceps (AF), the foil template (FT) is introduced into the site of the prepared defect to confirm that an accurate template has been sized.

The debrided lesion is then dried with a gauze patty, and the curved raspatory tool from the Chondrectom set is used to measure the size of the lesion (Fig 6). These approximate measurements are then transferred to the foil template included within the Chondro-Gide package and cut to size. Small artery forceps are used to introduce the cut template into the defect bed, and the size and shape of the template are checked for adequacy. It is important to size the foil template to match the defect bed as accurately as possible, as this greatly influences the subsequent adequacy of defect filling (Fig 7).

**Fig 8:**
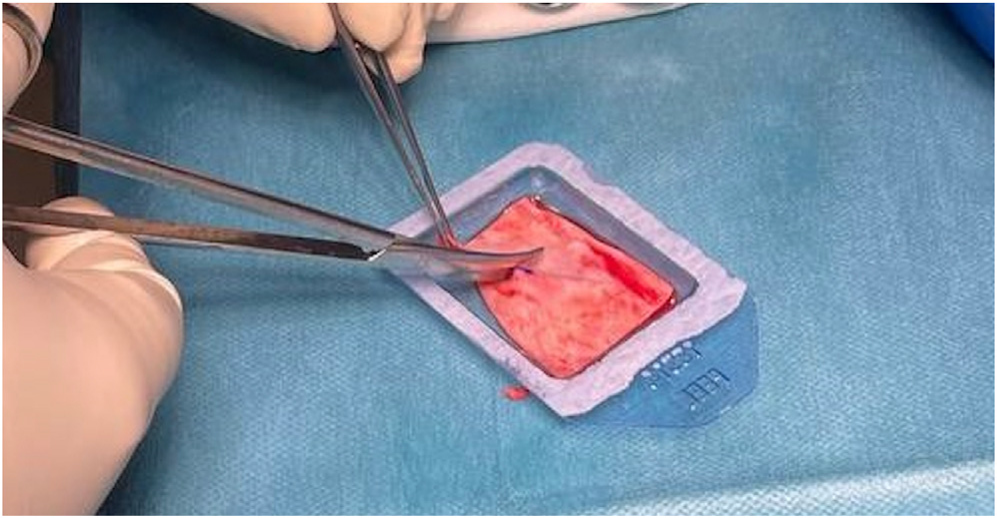
Using a pair of Metzenbaum scissors and handling the graft with atraumatic forceps, the graft is carefully cut to size, ensuring only sharp cuts are made to avoid fraying of the graft.

The BMAC-soaked Chondro-Gide is then cut exactly to the shape of the finalized foil template using a 15-blade scalpel and straight Metzenbaum scissors. At this point, the marking indicating the articular surface of the graft is re-shaded with a sterile skin marker to ensure visibility within the arthroscopic field (Fig 8).

### Cartilage Repair

**Fig 9:**
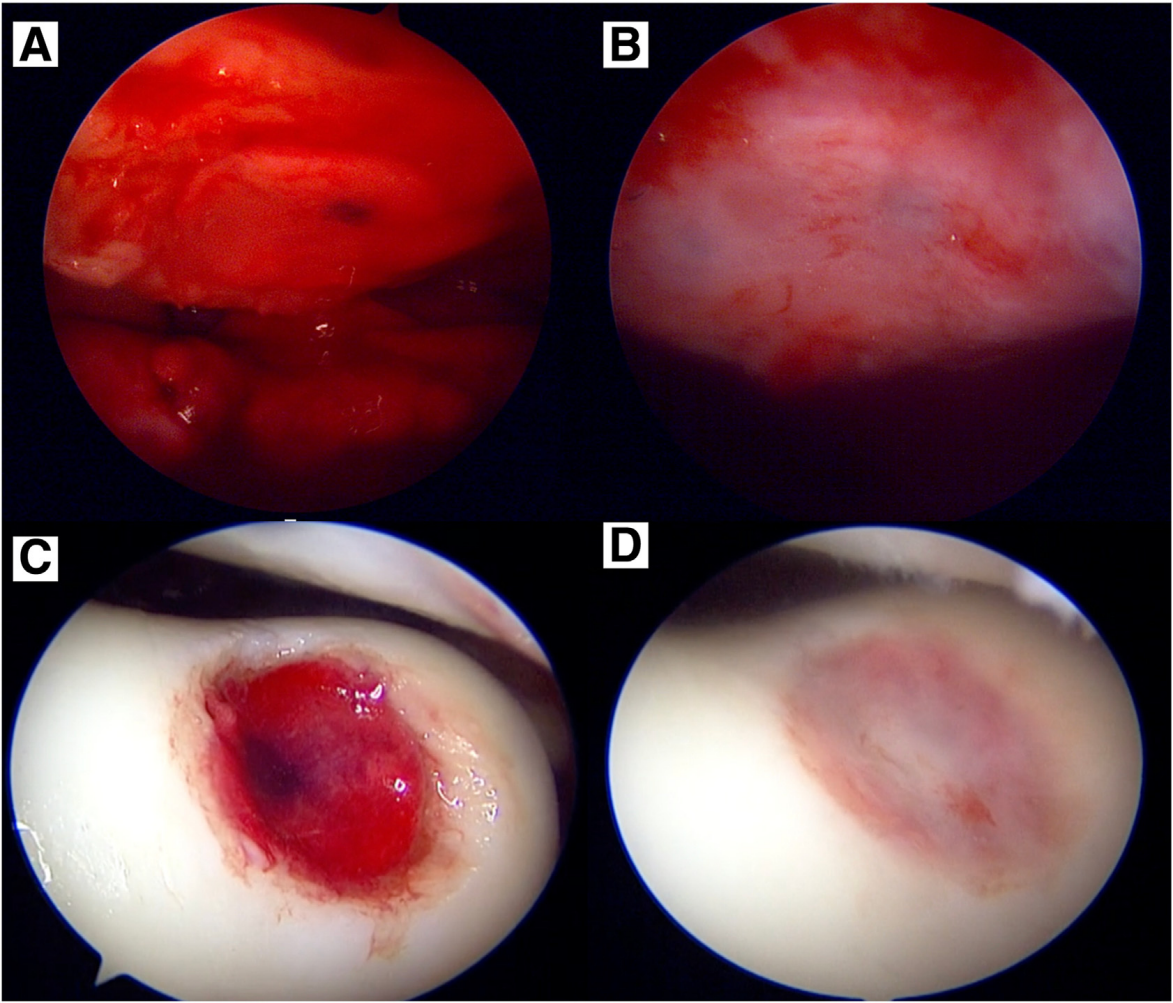
Graft in situ for the right knee patella lesion (A) and trochlear lesion (C) with Tisseel glue applied. After 5 minutes, the Tisseel glue has dried; and the patella graft (B) and trochlear graft (D) are being tested under saline (wet) arthroscopy to ensure stability.

The prepared graft is carefully introduced into the arthroscopic field with a pair of small artery forceps gently grasping the apex of the graft so as not to traumatize the scaffold matrix. At this point, direct visual confirmation of the articular surface marking is obtained to ensure the graft has been positioned correctly. The graft is placed loosely onto the lesion bed and the curved raspatory instrument is reintroduced to assist in smoothing and tamping the graft onto the lesion bed. Tisseel (Baxter, Deerfield, IL), a fibrin sealant, is then liberally applied to the surface of the graft in layers and left to dry for at least 5 minutes, as per manufacturer’s instructions. After drying, the Tisseel layer is palpated with the curved raspatory to check for contact bleeding, which signifies an inadequately dried layer. If contact bleeding is present, further time is allowed for Tisseel drying. Once the layer is stable to palpation with an absence of contact bleeding, the repair is considered complete, and the stability of the repair must now be evaluated. All devices are removed from the working field, and the knee is passively ranged through maximal flexion and extension for a total of 10 cycles of motion. Dry arthroscopy is then resumed, and the repair is checked visually for displacement or dislodgement. This process of evaluation is subsequently repeated under saline arthroscopy (Fig 9).

### Postoperative Protocol

The knee is then fitted with an angle brace and locked in extension, with continuous passive motion instituted from the first postoperative day. The prescribed ranges of motion and weight-bearing protocol differs based on location of the repair and is summarized in Tables 1 and 2 (Video 1).

**Table 1:** Postoperative Rehabilitation Protocol for Tibiofemoral Lesions

Tibiofemoral Lesions	Week 0-2	Weeks 3-4	Weeks 5-6	Weeks 7-8	Week 9 Onwards
Knee flexion permitted in locking angle brace	Up to 30°	Up to 60°	Up to 90°	Full range with brace worn	Full range without brace
Weight-bearing status	Non–weight-bearing	Non–weight-bearing	Protected weight-bearing in full extension	Full weight-bearing in full extension	Full weight-bearing with quads strengthening

**Table 2:** Postoperative Rehabilitation Protocol for Patellofemoral Lesions

Patellofemoral Lesions	Week 0-2	Weeks 3-4	Weeks 5-6	Weeks 7-8	Week 9 Onwards
Knee flexion permitted in locking angle brace	Up to 30°	Up to 60°	Up to 90°	Full range without brace	Full range without brace
Weight-bearing status	Non–weight-bearing	Protected weight-bearing in full extension	Full weight-bearing in full extension	Full weight-bearing with quads strengthening	Full weight-bearing with quads strengthening

## Discussion

We present our single-staged all arthroscopic cartilage repair using BMAC-augmented Chondro-Gide to treat full-thickness, critically sized articular defects of the knee. Additionally, we demonstrate that this technique is amenable in utility for both tibiofemoral and patellofemoral lesions. Table 3 summarizes the tips and pearls from our experience.

**Table 3:** Pearls and Pitfalls for Using Our Technique

Tips and pearls for using an all-arthroscopic approach to AMIC Plus	
Patellar lesions	Direct visualization of the lesions is crucial before chondrectomy.
	- Consider using a 70° arthroscope
	- Place the working portals closer to the joint line to allow for a better lever arm when debriding
Dry arthroscopy	A dry arthroscopic environment will greatly assist in graft implantation
	- Insufflate the joint with carbon dioxide gas at an adequate pressure
	- Introduce a metal suction tip on passive suction to the areas of fluid accumulation within working field
	- Allow the positive pressure differential within the joint to evacuate fluids through the passive metal suction tip
Scaffold adhesion	It is imperative to keep the cartilage defect bed as dry as possible when attaching the scaffold
	- Remove any gross liquid collection with the aforementioned metal suction tip setup
	- Tamp the defect bed dry with arthroscopic surgical patties

Pitfalls to avoid when using an all-arthroscopic approach to AMIC Plus
- Ensure blood pressure is tightly controlled during the implantation process, as uncontrolled hypertension will result in excessive subchondral and joint space bleeding, obscuring vision during dry arthroscopy. - Aim for complete removal of loose cartilage bodies after debridement/chondrectomy by thoroughly washing the knee joint before transitioning to dry arthroscopy.

The all-arthroscopic approach, with templating and augmentation of orthobiologics, is the main advantage of the technique that we present in this study. Conventionally, the repair of critically sized articular defects with Chondro-Gide is performed through a mini-open arthrotomy due to the need for templating the Chondro-Gide membrane scaffold, for a customized fit. Such approaches, although excellent for achieving exposure, direct visualization, and manipulation of intra-articular structures, carry the standard increased risks of arthrotomy over arthroscopy, and are often associated with longer postoperative recovery periods, more significant postoperative pain.^4^ Our technique allows for minimal violation of soft tissues, a precise fit of the membrane scaffold in the defect, as well as providing advantages of orthobiologics augmentation. A limitation of this technique would be addressing patella cartilage defects with associated bone loss, where bone grafting before placement of the membrane scaffold is required. In the arthroscopic approach, this would be technically challenging, and the mini-open approach would still be recommended.

Currently, there exists high-quality evidence supporting the short- and long-term clinical efficacy of Chondro-Gide in the treatment of articular defects of the knee, particularly when used in conjunction with BMAC.^5^^,^^6^ Steinwachs et al.^6^ displayed that for cartilage lesions with a mean size of 4 cm^2^, the utility of Chondro-Gide resulted in postoperative pain relief and functional improvement that was not only clinically significant but maintained for up to 3 years’ post-repair. In addition, among the 12 studies reviewed by the authors, no cases of treatment-related adverse events were noted. Consequently, this slate of evidence in support of the safety and efficacy of Chondro-Gide has led to the adoption of the AMIC and AMIC Plus procedures as the recommended standard of care for critically sized articular cartilage lesions by international consensus bodies.^7^

In conclusion, the AMIC Plus procedure for critically sized articular defects of the knee results has been displayed to have a good clinical safety and efficacy profile. Hence, the addition of an all-arthroscopic approach to performing this procedure would allow a cartilage surgeon to deliver the current standard of care for cartilage defects through a minimally invasive surgical approach, with minimal disruption to knee capsule and extensor mechanism integrity.

## Supplementary Data

ICMJE author disclosure forms

Video 1 Shown is our technique of an all-arthroscopic single-staged cartilage repair procedure with an acellular collagen I/III bilayer scaffold (Chondro-Gide) augmented with bone marrow aspirate concentrate. We demonstrate our technique first in a trochlear lesion, followed by medial femoral condyle and patellar lesions.

## References

[1] Ow Z.G.W., Cheang H.L.X., Koh J.H. (2022). Does the choice of acellular scaffold and augmentation with bone marrow aspirate concentrate affect short-term outcomes in cartilage repair? A systematic review and meta-analysis. Am J Sports Med.

[2] Mall N.A., Harris J.D., Cole B.J. (2015). Clinical evaluation and preoperative planning of articular cartilage lesions of the knee. J Am Acad Orthop Surg.

[3] Hevesi M., van Genechten W., Krych A.J., Saris D.B.F. (2021). The sound of cartilage repair: The importance of using pitch and volume cues in cartilage restoration surgery. Arthrosc Tech.

[4] Peres L.R., Marchitto R.O., Pereira G.S., Yoshino F.S., de Castro Fernandes M., Matsumoto M.H. (2016). Arthrotomy versus arthroscopy in the treatment of septic arthritis of the knee in adults: A randomized clinical trial. Knee Surg Sports Traumatol Arthrosc.

[5] de Girolamo L., Schönhuber H., Viganò M. (2019). Autologous matrix-induced chondrogenesis (AMIC) and AMIC enhanced by autologous concentrated bone marrow aspirate (BMAC) allow for stable clinical and functional improvements at up to 9 years follow-up: Results from a randomized controlled study. J Clin Med.

[6] Steinwachs M.R., Gille J., Volz M. (2021). Systematic review and meta-analysis of the clinical evidence on the use of autologous matrix-induced chondrogenesis in the knee. Cartilage.

[7] Niemeyer P., Albrecht D., Aurich M. (2022). Empfehlungen der AG Klinische Geweberegeneration zur Behandlung von Knorpelschäden am Kniegelenk. Z Orthop Unfall.

